# Reply to “Lung Ultrasound in the Diagnosis of Pleural Tuberculosis”

**DOI:** 10.31662/jmaj.2026-0266

**Published:** 2026-07-31

**Authors:** Yuki Ohnishi, Yasuhiro Suyama, Kenichi Nakamura

**Affiliations:** 1 Department of Family Medicine, SUNY Upstate Medical University, Syracuse, New York, USA; 2 Department of Rheumatology, NTT Medical Center Tokyo, Tokyo, Japan; 3 Department of General Internal Medicine, Aso Iizuka Hospital, Fukuoka, Japan

**Keywords:** pleura, tuberculosis, tuberculous pleurisy, ultrasound

We truly appreciate Dr. Chang’s valuable comments regarding our recently published image report on sonographically detected tuberculous pleurisy ^(1)^. Their thoughtful review meaningfully contributes to a better understanding of the key sonographic pleural features of pulmonary tuberculosis (PTB).

The French intensivist Dr. Daniel Lichtenstein began studying lung ultrasound (LUS) artifacts in the mid-1990s and described their application to lung pathology ^(2)^. He demonstrated that these artifacts can provide diagnostically useful information, thereby challenging the conventional belief that the lung cannot be accurately evaluated using ultrasound due to such artifacts.

While our original report focused on the use of ultrasound for the diagnosis of tuberculous pleurisy, LUS has been increasingly explored not only for diagnosis and screening but also for monitoring treatment response in PTB. Weber et al. ^(3)^ investigated whether LUS could serve as a noninvasive, radiation-free, and accessible modality for monitoring treatment response in PTB, particularly in settings with limited access to conventional follow-up methods. This approach may be especially valuable in resource-limited settings.

As Dr. Chang noted, LUS is operator dependent and insufficient as a standalone tool for PTB. Current evidence supports its use only as an adjunct, interpreted alongside clinical and radiologic findings. For instance, Bigio et al. ^(4)^ reported that subpleural nodules and lung consolidation were the sonographic findings with the highest sensitivities in adults, ranging from 72.5% to 100.0% and from 46.7% to 80.4%, respectively, although specificity data were limited and standardized protocols were lacking. A recent systematic review and meta-analysis by Hassankhani et al. ^(5)^ demonstrated that conventional LUS is a sensitive adjunct for PTB detection but lacks sufficient specificity and likelihood ratios to function as an independent diagnostic tool. The authors concluded that further development of standardized protocols and improved implementation strategies are necessary to enhance its diagnostic performance ^(5)^.

Dr. Chang’s insightful comments contribute meaningfully to the ongoing discussion on the clinical application of ultrasound in PTB, a condition of global clinical importance. As emphasized in our imaging report, the high sensitivity of ultrasound may enable the detection of thoracic pathology even in patients presenting with atypical symptoms, such as abdominal pain due to referred pain. The identification of pleural or peripheral pulmonary abnormalities during ultrasonography may offer important diagnostic clues to tuberculous pleurisy and highlights the need for awareness of lung findings during routine ultrasound evaluation.

## Article Information

### Author Contributions

Yuki Ohnishi drafted the manuscript. Yasuhiro Suyama and Kenichi Nakamura reviewed and edited the manuscript.

### Conflicts of Interest

None
